# Advances in the Production of Biomaterials through Kombucha Using Food Waste: Concepts, Challenges, and Potential

**DOI:** 10.3390/polym15071701

**Published:** 2023-03-29

**Authors:** Anelise Leal Vieira Cubas, Ana Paula Provin, Ana Regina Aguiar Dutra, Cláudia Mouro, Isabel C. Gouveia

**Affiliations:** 1 Environmental Science Master’s Program, University of Southern Santa Catarina (Unisul), Avenida Pedra Branca, 25, Palhoça 80137270, SC, Brazil; 2 FibEnTech R&D—Fiber Materials and Environmental Technologies, University of Beira Interior, Rua Marquês d’Avila e Bolama, 6201-001 Covilhã, Portugal

**Keywords:** biomaterial, bacterial cellulose, kombucha, food waste, sustainability

## Abstract

In recent years, several researchers have focused their studies on the development of sustainable biomaterials using renewable sources, including the incorporation of living biological systems. One of the best biomaterials is bacterial cellulose (BC). There are several ways to produce BC, from using a pure strain to producing the fermented drink kombucha, which has a symbiotic culture of bacteria and yeasts (SCOBY). Studies have shown that the use of agricultural waste can be a low-cost and sustainable way to create BC. This article conducts a literature review to analyze issues related to the creation of BC through kombucha production. The databases used were ScienceDirect, Scopus, Web of Science, and SpringerLink. A total of 42 articles, dated from 2018 to 2022, were referenced to write this review. The findings contributed to the discussion of three topics: (1) The production of BC through food waste (including patents in addition to the scientific literature); (2) Areas of research, sectors, and products that use BC (including research that did not use the kombucha drink, but used food waste as a source of carbon and nitrogen); and (3) Production, sustainability, and circular economy: perspectives, challenges, and trends in the use of BC (including some advantages and disadvantages of BC production through the kombucha drink).

## 1. Introduction

Many researchers have drawn attention to the development of sustainable biomaterials obtained from renewable sources. Approximately 100–150 billion tons of cellulose are synthesized naturally every year globally, making it one of the most abundant and recognized materials on Earth [1,2]. In addition, cellulose is obtained from plants and recognized for being an organic and biodegradable polymer [2]. For many years, natural biomaterials have benefited human civilization. With advances in materials science, new ways of manufacturing, processing, and functionalizing biomaterials are being developed [3].

According to Gupte et al. (2021) and collaborators, the world is moving towards more sustainable and environmentally friendly materials. The production of biomaterials, such as nanocellulose, on a large scale can be crucial for the replacement of plastics-based fossil fuels. The authors also note that the production of nanocellulose from plants can lead to mass deforestation and will require large amounts of fresh water for reforestation [4].

The inclusion of living biological systems has led to the creation of strategies and solutions. This area of study emerged from the understanding that there is a need to develop new sustainable materials that are not a source of environmental pollution. Important opportunities have arisen in the biotechnology and biomanufacturing sectors [5].

One of the best-known biomaterials is bacterial cellulose (BC). In 1886, the pioneer researcher Brown described, for the first time, the strain *A. xylinum*, naming it a ‘vinegar plant’ from the presence of a gelatinous mass on the surface of the culture fluid. In-depth studies have since been carried out to understand cellulose biosynthesis by *A. xylinum* [1,6].

Behera et al. (2022) and Tapias et al. (2022), state that cellulose contributes ≅ 1.5 trillion tons of biomass every year. Its plant sources come from hemp, cotton and flax, among others, and microbial sources, such as bacteria or algae. Microorganisms, such as *Acetobacter*, *Agrobacterium*, *Azotobacter*, and *Rhizobium,* are known for their BC production. The bacterium *Acetobacter xylinum* has received great attention by researchers for its cellulose production capacity [7,8]. Tran et al. (2021) and Gupte et al. (2021) also point out that *Acetobacter* was further classified as *Gluconacetobacter* (glucose in cellulose) or *Komagataeibacter* (named after a Japanese microbiologist, due to his contribution to the systematics of AAB) [4,9].

One way to produce BC is through the probiotic drink kombucha. Originating from northeast China [10,11], it is obtained from the fermentation of a sweetened tea mixture with mixed cultures of acetic acid bacteria and yeasts for a period of 10 to 14 days [7,8]. During fermentation, microorganisms consume sucrose as the main carbon source. The tea extract provides the nitrogen source and in the presence of oxygen, the SCOBY produces organic acids, carbon dioxide, and a cellulosic biofilm [12,13].

Combined with other food ingredients, BC has high potential value due to its superior water holding capacity, high purity, and low-calorie dietary fiber, making it an edible biopolymer. BC is known to be a fiber-rich natural food that offers many health benefits and a reduction in the risk of chronic diseases, such as diabetes, obesity, and cardiovascular disease. In 1992, BC was recognized as safe by the Food and Drug Administration [3].

The production of kombucha is usually done by the static culture method. In the container used for cellulose growth, physical, chemical, and biological factors are considered for its formation. The existence of the agitated mode of cultivation can also be used to create kombucha, but is not the most used form due to high turbulence and shear stress in the cells. This can cause mutations in the cells, making them negative [11].

The BC produced by kombucha has received a lot of attention due to its excellent mechanical and physicochemical properties, and is exploited for different applications, such as food, textile, and environmental. In addition to its unique physicochemical properties compared to plant cellulose, 79.2 tons of BC in 500,000 L can be produced in only 22 days; while in 1-hectare of land, only 80 tons of cellulose are produced by the plants during 7 years [1,7]. However, the lack of information about the feasibility of the process often makes it difficult to manufacture large-scale kombucha-based pulp [7].

The use of agricultural residues can be a low-cost and sustainable means of production for the creation of BC. Published research reports several agricultural sources for the production of BC with oat husk; grape extract; cherry husk coffee; and fruits, such as pineapple, pomegranate, melon, watermelon, tomato, and orange, among others [3,14]. According to Kumar et al. (2022), the volume of food waste generated globally is 1.3 to 1.4 billion tons and is expected to increase to 2.6 billion tons by 2025 [15]. In this sense, the production of BC can contribute to the minimization of food waste, as these are rich in carbon source, food for acetic bacteria, and therefore, can be used as raw material [16].

This article will discuss the issues regarding the production of BC through food waste, the sectors using BC, the products developed, the issues of sustainability, and the circular economy behind these studies. Finally, the perspectives, challenges, and trends for the use of BC will be presented as reported by the researchers and their published articles.

## 2. Methods

**Figure 1:**
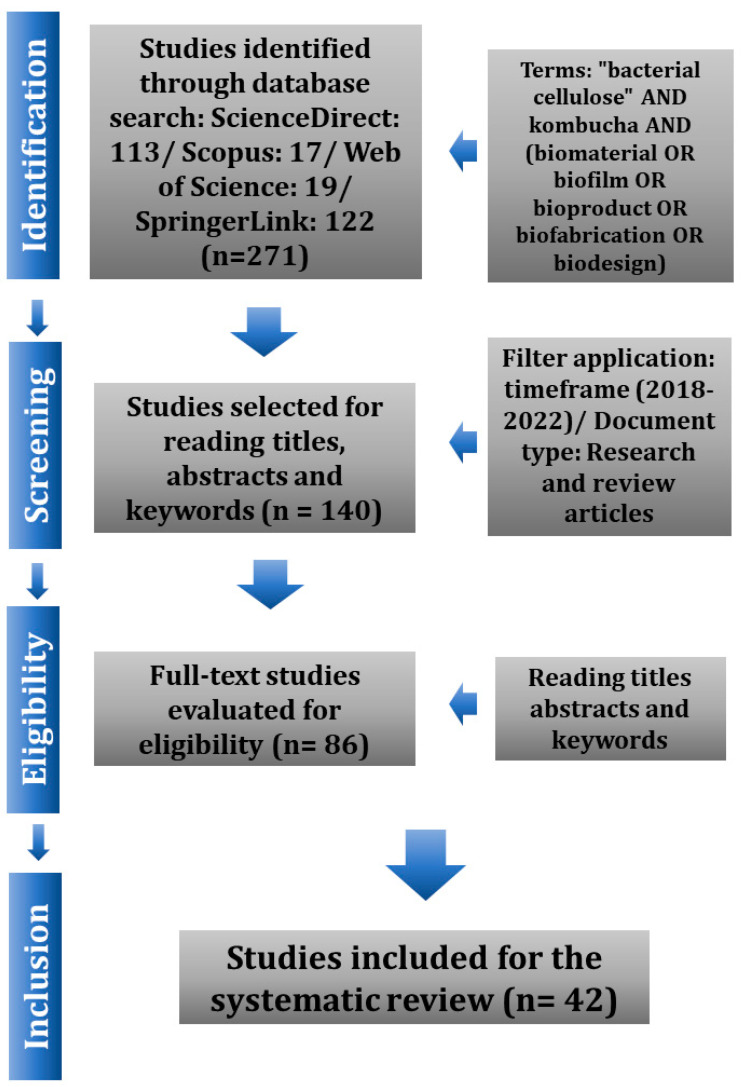
Exemplification of the method used for the selection of research articles. Source: authors, 2022.

A systematic literature review was used to search for articles that discussed the analysis and advances in the production of biomaterials through kombucha using food waste. The review filtered through research and review articles published, between 2018 to 2022, in peer-reviewed academic journals, excluding books, book chapters, programs, and projects. The databases used were ScienceDirect, Scopus, Web of Science, and SpringerLink. Seven search terms were used: “*bacterial cellulose*” AND *kombucha* AND (*biomaterial* OR *biofilm* OR *bioproduct* OR *biofabrication* OR *biodesign*). Figure 1 exemplifies the method used to select articles for this review.

Forty articles were found using search terms, document type, time interval, abstract, and keywords. The study provides the following elements of analysis to contemplate the results and contents researched by this systematic literature review:

(a) Results: survey of the data that appeared in the forty articles read in full.
(b) Discussion.
(c) Production of BC through food waste.
(d) Research, sectors, and products: the use of BC and the areas of study.
(e) Production, sustainability, and circular economy: Perspectives, challenges, and trends in the use of BC.

In addition to the scientific literature review, a search was conducted on Google Patents with the purpose of investigating existing records related to the production of CB using kombucha. The search terms used were “bacterial cellulose” AND “kombucha”, and the filter “Status Active” was selected. It is noteworthy that the purpose of patents in the scientific field is to protect the intellectual property of inventions or discoveries made by university researchers, professors, and students. Furthermore, patents encourage research and the development of new technologies by offering financial incentives and recognition to researchers.

## 3. Results

**Figure 2:**
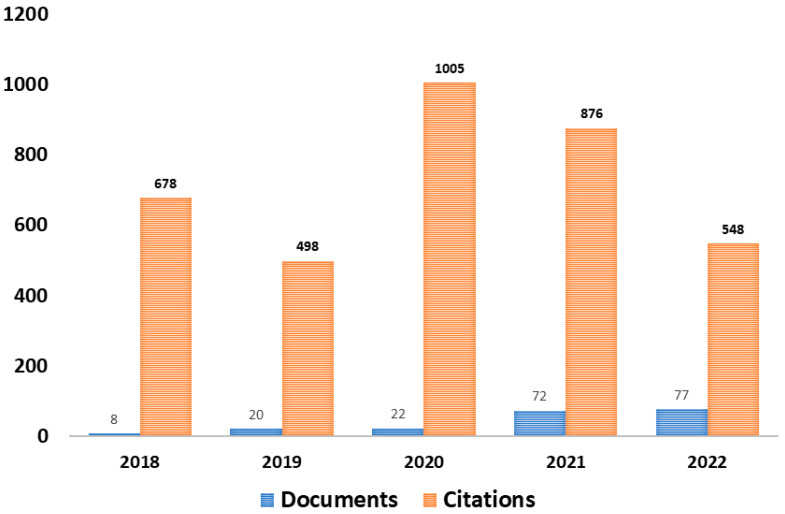
Number of documents and citations in the databases using the search terms and filter between 2018 and 2022. Source: authors, 2022.

When searching for the terms “*bacterial cellulose*” AND *kombucha* AND (*biomaterial* OR *biofilm* OR *bioproduct* OR *biofabrication* OR *biodesign*), 113 results were found from the ScienceDirect database, 17 results from Scopus, 122 results from SpringerLink, and 19 results from the Web of Science. Subsequently, when the “Document type” and “Time clipping” filters were applied, the search results were narrowed, leaving 68 from the ScienceDirect database, 14 results from Scopus, 43 results from SpringerLink, and 15 results from the Web of Science. Figure 2 shows the number of documents and citations in the databases using the search terms and time filter between 2018 to 2022.

After reading the titles, abstracts, and keywords, 44 articles were left from the ScienceDirect database, 11 articles from Scopus, 19 articles from SpringerLink, and 12 articles from the Web of Science, with a resulting total of 86 research articles. After reading the articles in full and checking for duplicity between the databases, 44 articles were excluded, totaling 42 articles for the writing of this systematic literature review article.

**Figure 3:**
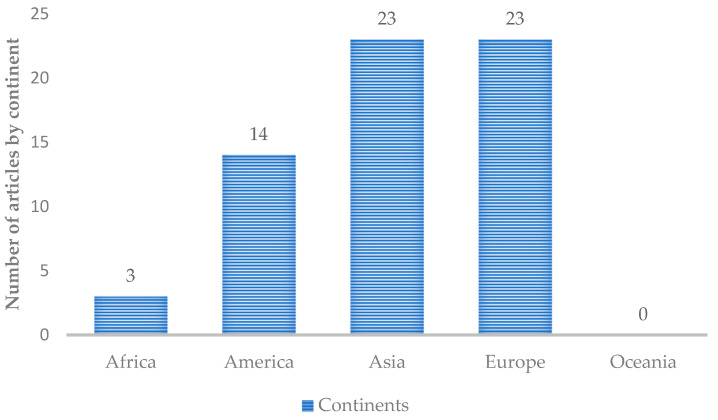
Graph showing the number of publications by continent. Source: authors, 2022.

The 42 research and review articles analyzed were in line with the theme “Advances in the production of biomaterials through the kombucha drink using food waste”. The documents were organized in a table (Table A1) with titles, authors, year of publication, country of origin of the research, area of expertise, and number of citations. Figure 3 shows the number of articles per continent.

**Figure 4:**
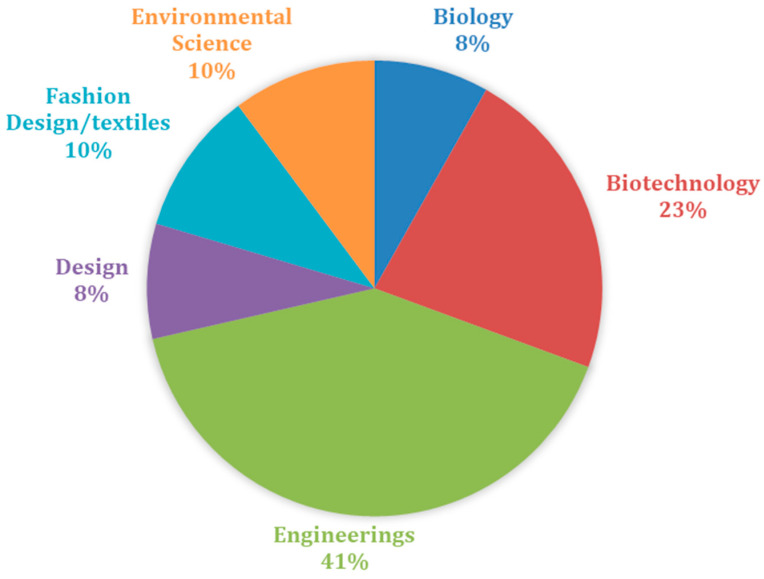
Analysis of the participation of the research areas of the 42 articles that make up the portfolio. Source: authors, 2022.

The continents that participated the most in the analyzed surveys were Asia with 23 participations, Europe with 23 participations, and America with 14 participations. India produced 11 articles and Brazil produced 10 articles. Figure 4 shows the research areas that had the most participation in the 42 articles read.

**Figure 5:**
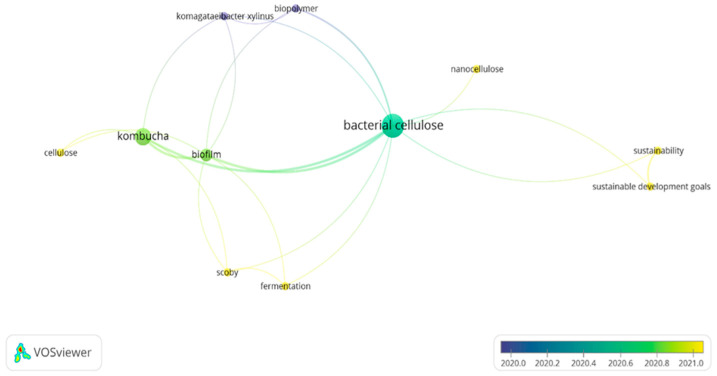
Co-occurrence among the most used terms among the 42 research articles. Source: authors, 2022.

As shown in Figure 4, the research areas that were most expressive in the documents were linked to engineering, biotechnology, environmental sciences, design, biology, fashion, and textile design. However, other areas of research can be noted in Table A1. When analyzing the terms that had the highest co-occurrence among the 42 research articles, the terms found were: “bacterial cellulose”, “kombucha”, and “biofilm”, as can be seen in Figure 5.

To create Figure 4, the VOS viewer software was used, which is widely used in systematic and bibliometric reviews, mapping the co-occurrence of terms, citations, authors, among others. For this, the 42 articles from the final portfolio of this review were used. According to Figure 4, the terms “bacterial cellulose”, “kombucha”, and “biofilm” had a greater use in 2020, with discussions associated with sustainability issues and relationships with the Sustainable Development Goal occurring between 2020 and 2021.

It should be noted that the BC has great potential to contribute to the fulfillment of the SDGs. One can relate SDG 12 (Responsible consumption and production), which aims to ensure responsible consumption and production patterns, mainly with regard to targets 12.5, which aims to reduce waste generation by 2030, and 12.c, which aims to rationalize subsidies inefficient to fossil fuels. Recalling here that biotextiles can be promising as substitutes for materials derived from petroleum, such as polyester [17].

Additionally, SDG 9 is noteworthy (Industry, innovation, and infrastructure), as it aims to build resilient infrastructures, promote inclusive, and sustainable industrialization and fosters innovation. BC production can contribute to goals, such as 9.4, which aims to modernize and make industries more sustainable through cleaner processes and technologies, and 9.5, which aims to strengthen research, encouraging innovation, especially in developing countries [17].

With the findings of the 42 articles, 3 topics were narrowed down for discussion. Table 1 presents the topics and the respective authors who contributed.

According to Table 1, it was possible to narrow the subject addressed “Advances in the production of biomaterials through the kombucha drink using food waste: concepts, challenges and potentialities” into three thematic axes:

(1) Production of BC through food waste: this topic addresses which types of food waste researchers have used in their studies.
(2) Research, sectors, and products—the use of BC and areas of study: this topic addresses which research areas have enjoyed the benefits of BC as a promising biomaterial for the development of products and services.
(3) Production, sustainability, and circular economy—perspectives, challenges, and trends in the use of BC: this topic addresses the points raised by researchers in relation to sustainability, challenges, and trends in the use of BC.

## 4. Discussion

### 4.1. Production of Bacterial Cellulose through Food Waste

Food waste generated by the food industry has received a lot of global attention in recent years. Studies show that in Latin American and Caribbean countries, organic waste represents the largest proportion of generated waste (≅50%). However, studies have found that food waste is a precious bio-resource that can be used to develop many useful chemicals, materials, and fuels. It was observed that food residues have great potential as a raw material (primary or secondary) to produce biopolymers by extraction or fermentation in an attempt to minimize the negative environmental impact, the growing concern for sustainability has encouraged research into biodegradable polymers [39].

The production of kombucha drinks has started to grow. It already has substantial market value and is expected to increase in the future. Kombucha beverage industries often discard BC as waste or use a small portion as inoculum for further fermentation. However, it is known that BC can be used as a cheaper source of cellulose for industrial purposes [7].

For the production of BC through the kombucha fermentation process, several foods can be used, from different herbal infusions, such as black tea, green tea, yerba mate, lavender, oregano, and fennel [8]. Some medicinal and flavoring plant leaves were also used for fermentation, such as lemon balm, thyme, and peppermint. It was discovered that fermentation took place in less days compared to the traditional method [11].

Some researchers also brought in other alternative raw materials for the fermentation of kombucha drinks, such as milk; coffee; cactus juice; grape juice; snake fruit; rooibos tea; black goji berry; African mustard leaves; black carrot juice; cherry blonde; blackthorn red raspberry; acerola [18]; coconut cream; red wine; beer [2]; pecan shells; soapberry; sugar cane syrup; hydrolyzate of pomace, pineapple, and pear peel; pomace; sweet sorghum; cellar residues; waste water from oil mills; miscanthus; and sweet potato [19].

El-Gendi et al. (2022) also reported for the production of BC in: spruce hydrolyzate, hot water wood extracts, pineapple agro-industrial residues, citrus juices, rotten fruits, cotton-based textile residues, beet and sugar cane molasses, residues of food processing, wine fermentation waste broth, candied jujube processing industry waste water, waste streams and by-products from biodiesel and confectionery industries, acetone-butanol-ethanol fermentation waste water, citrus peel, hemicellulose, konjac, rice husk, wheat straw, maple syrup, coffee cherry peel, dried olive mill residue, residual brewer’s yeast, and date syrup [20].

Nie et al. (2022) performed a study with three new strains that were isolated from traditional rice vinegar. *Acetobacter pasteurianus* MGC-N8819 exhibited a relatively high BC yield of 6.6 g/L on Hestrin-Schramm (HS) medium [19]. The researchers also carried out tests with the agricultural lotus rhizome. The combination of alpha-amylase and glucoamylase treatments effectively increased the release of reduced sugar from rhizome by-products, achieving a BC yield of 2.1 g/L and BC productivity of 0.26 g/(L·day). Nie et al. (2022) also point out that the lotus (Nelumbo nucifera Gaertn.) is an aquatic plant belonging to the *Nelumbonaceae* family that has historically been cultivated in East and Southeast Asia. In China, the annual production of lotus rhizome is approximately 1.2 × 10^7^ tons [19].

Sugarcane molasses is also a by-product of sugar production that has large amounts of fermentable sugars, such as fructose, sucrose, and glucose. It has been considered the most economical carbon source in the fermentation industry [14]. According to Machado et al. (2018), molasses also has sources of nitrogen and vitamins, which are important in optimizing BC production. Brazil represents the top rank of sugarcane producing countries of sugar, including the production of sugar and ethanol [14].

Fernandes et al. (2020) also highlighted that to meet the growing demands for renewable and sustainable biomaterials derived from biomass, it is interesting to use BC food waste [21]. In their studies, they observed the use of an in situ modification to develop b film with graphene oxide using sugarcane residues as a carbon source. As a result, the film exhibited high crystallinity and surface area, a high degree of polymerization, and improved mechanical properties [21].

Revin et al. (2018) developed a research on cost reduction for obtaining BCthrough acid by-products of the alcohol and dairy industries [22]. The studies showed that the greatest accumulation of BC (6.19 g/L) occurs in fine wheat vinasse for 3 days of cultivation under dynamic conditions, which is almost 3 times higher than in the standard Hestrin and Schramm medium (2.14 g/L). They also highlighted the use of whey as a nutrient medium, which allowed obtaining 5.45 g/L of BC under similar cultivation conditions [22].

Greser and Avcioglu (2022) studied the use of grape, apple, and apple vinegar as potential BC producers [23]. The strains isolated from grape vinegar were identified as *Komagataeibacter maltaceti,* and the strain isolated from prickly apple vinegar was identified as *Komagataeibacter nataicola*. They were found as 8% dextrin, 1.5% (peptone + yeast extract), and 10% inoculation amount at pH 6.0 with a productivity rate of 1.15 g/d/L, yield of 8.06%, dry weight of 6.45 g/L for *K. maltaceti*, 10% maltose, 1% (peptone + yeast extract), and 10% inoculation amount at pH 6.0 with a productivity rate of 0.96 g/L/d, 5.35% yield, and 5.35 g/L dry weight for *K. nataicola*. The BC obtained from the strains of *K. maltaceti* and *K. nataicola* was more than 2.56- and 1.86-fold when compared to the BC obtained from the HS medium. As a conclusion of the study, it was observed that both species isolated from *Komagataeibacter* can be used in the production of biopolymers from residues containing complex sources of carbon [23].

Table 2 displays the articles and objectives of each study related to the waste used for kombucha productions.

Table 2 presents 12 studies related to BC production. Out of the 12 studies, 8 directly address the production of BC using kombucha or microorganisms isolated from kombucha tea. The other 4 studies describe various methods of BC production but mention the kombucha beverage as one of the ways to produce BC.

In addition to the published scientific research, a patent search was also conducted with the aim of registering some inventions developed by the academic community and patented. Table 2 shows the registration of 5 patents for the production of BC using kombucha.

According to Table 3, out of the 5 patents, 4 are from Donghua University in China. It is worth noting that kombucha has reports of its origin in China around 200 BC and is known for its medicinal and probiotic properties [24]. In the first patent, CN101985641B, the invention presents a method for obtaining BC from wheat straw waste, which is a cheap source of carbon and helps to reduce environmental pollution. The process involves several steps, including pre-treatment of the straw in an ionic liquid, enzymolysis, and cultivation of cellulose-producing bacteria extracted from kombucha or tea fungus (*Acetobacter xylinum*).

The second patent, CN102533904B, presents a method and device for rapidly producing large-scale BC composite materials involving the cultivation of a bacterial strain in a liquid medium (one of the modalities using 300 mL of kombucha) and transfer to a biological reactor containing structure material for cultivation disturbance. The device is simple, low-cost, and highly-automated, allowing for high production efficiency and obtaining a BC composite material applicable in various areas.

The third patent, CN102242166B, describes a method for preparing BC using inulin as a carbon source. The process involves hydrolysis of inulin, addition of a nitrogen source, cultivation of the bacterial strain, and obtainingBC. The method is advantageous for its low cost and wide range of raw material sources. The inventors report using Gluconacetobacter xylinus or kombucha tea fungus.

The fourth patent, EP3121265B1, aims to isolate bacterial strains that produce nanostructured cellulose and increase their production rate. According to the inventors, one of the strains used was B17 isolated from kombucha tea cell culture.

Finally, the fifth patent, CN102250983B, includes hydrolysis of Jerusalem artichoke to obtain a hydrolyzed solution, which is used as a carbon source for fermentation medium along with a nitrogen source. The seed liquor of the bacterial strain producing cellulose is added to the fermentation medium to produce BC in 3–23 days. According to the inventors, the technology has wide sources of raw material, low cost, and good prospects for application in the field of BC production. The invention includes Gluconacetobacter xylinus or red tea fungus (kombucha) as part of its content.

### 4.2. Research, Sectors, and Products: The Use of Bacterial Cellulose and Areas of Study

According to Subbiahdoss et al. (2022) and Sederavičiūtė et al. (2019), studies show that biofilm-forming bacteria (solid–liquid, liquid–liquid, and liquid–air) can impact the healthcare, pharmaceutical, food, and oil recovery industries [25,26]. as Additionally, having properties, such as high crystallinity, biocompatibility, non-toxicity, and high porosity, they make it a suitable material for technological applications in bioplastics, bioenergy, food fortification, and packaging [11].

As mentioned earlier, one of the ways of producing BC is through the fermented drink kombucha. It should be noted that in the production process of kombucha, the biofilm is considered a by-product and, in most cases, is discarded [8]. However, several studies on kombucha SCOBY are being carried out to explore all possibilities of how to reuse this cellulose as a suitable raw material in areas, such as food technology, biomaterial preparation, environmental biotechnology, and fashion and textile industries [8,11].

Due to the BC biofilms derived from the kombucha beverage having good mechanical, physical, chemical, and biological properties, such as tensile strength, adsorption capacity, high melting temperature, and biodegradability, among others, BC can be used in several sectors that use biomaterials [7,24]. BC membrane is considered renewable, ecologically correct, sustainable, and cheaper, and has spread in different areas, such as paper production, filtration/separation, textiles, food industry, electronics, fire retardant, and biomedical devices [27].

In the healthcare sector, wound healing and burn recovery is an interesting area where the natural properties of BC can be utilized as BC has unique physicochemical characteristics [3]. Mirmohammadsadegh et al. (2022) developed a study using Pistacia atlantica fruit oil together with BC [28]. In traditional Persian medicine, the oil of this plant has been used to treat wounds, inflammation, and other ailments.

The main objective of the study was to analyze the chemical composition of *Pistacia atlantica* fruit oil and to study the wound healing and anti-inflammatory effects of BC absorbed by the oil in an in vivo burn model. The results showed that BC coated with *Pistacia atlantica* fruit oil may be a potential biosafe curative for wound treatment [28].

A study carried out by Navya et al. (2022), raised the use of BC in tissue engineering [29]. According to the authors, collagen is the main structural protein found in most tissues and organs, and is used in the construction of artificial skin substitutes, cell proliferation and functioning. It was then found that the significant water content, burst pressure, and crystalline nature along the fine fibrous structure of BC are similar to collagen, making BC useful for arterial grafting and vascular tissue engineering [29].

Kumar et al. (2022) worked on creating a more cost-effective antibacterial BC using alternative methods, such as tomato waste and Dracaena serrulata leaf extract, rather than traditional techniques involving metal nanoparticles or drug integration. The researchers emphasized the importance of producing environmentally friendly biomedical materials, which involve the reuse of waste to make production cheaper and to reduce the environmental risks associated with waste disposal and management [15].

Kamal et al. (2022) also worked on the creation of an antibacterial compound using BC with pomegranate peel extract (*Punica granatum* L.) (PGPE) for possible biomedical applications. To achieve this, the researchers produced BC using food waste in an economical way and impregnated the hydrogel with PGPE extract ex situ. The PGPE extract showed favorable antimicrobial properties against Gram-positive Staphylococcus aureus and Gram-negative *Escherichia coli* [16].

It should be noted that, although the biomedical applications are not directly produced through the kombucha drink, it was considered important to show these cases of biomedical production using food waste, as it contributes to the environment, becomes economically viable, and is a way of BC production. In this way, future research can continue to be developed in this sense [16]. It is important to note that the BC used for medical applications in the cited cases is not yet derived from kombucha in the examples mentioned in this section. However, there are already some cases of production using food waste to produce BC.

With rapid population growth, the global demand for goods and services increases rapidly, causing a greater demand for plastic packaging. This has increased the toxic effect of waste accumulation or incineration and attention has turned to biodegradable or bioplastic resources for packaging solutions [3]. Collaborators say that the global impact of the paper industry on deforestation has resulted in the search for alternative ways of producing paper. The characteristics necessary to reinforce degraded roles were identified in the BC.

Due to these excellent properties, BC finds wide applications in various fields, such as food packaging [30]. According to Gullo et al. (2018) BC modification can be widely used in food packaging to increase safety and shelf life [31]. Studies carried out regarding the antimicrobial effect have been demonstrated. The researchers added sorbic acid in mono and multilayers of BC against *E. coli* (K12-MG1655) and obtained positive results of its efficiency [31].

The biofilms were modified with a chitosan biopolymer by a simple immersion technique. The presence of chitosan in the film showed to have antibacterial property, good mechanical resistance, crystalline nature, and air resistance. The evaluation of the shelf life showed potential to be used in low cost active packaging, which is required by the packaging industry [32].

BC is also known for its outstanding characteristics as nanoscale materials. In addition, BC’s unique nanofibrous network architecture has significant effects on cell adhesion and proliferation. This property favors its application in 3D cell culture and tissue engineering applications, making it a promising material for highly biocompatible tissue structures [6].

Pillai et al. (2021) produced a sustainable kombucha BC membrane and it was acid treated to partially hydrolyze [33]. This controlled process improves its extrusion and forming capacity. Subsequently, BC was studied to assess its potential as a 3D printed scaffold. Two different cell lines (human dermal fibroblast cells and mice osteoblastic cells) were used to study cytocompatibility.

As a result, Pillai et al. (2021) found that both cell types showed good adhesion, growth and proliferation in pure and treated kombucha BC [33]. Research has indicated that the controlled partial hydrolysis of BC can make it suitable for 3D printing while maintaining its mechanical strength and cytocompatibility. The authors state that the sustainable microbial biopolymer proved possible to be used as a bioink for 3D bioprinting.

In the textile area, BC appears as an alternative for production and as a potential substitute for materials from fossil fuels [2]. One of the main reasons for these substitution strategies and product innovation initiatives is the increasing political pressure on textile manufacturing to reduce the impact [34].

Costa et al. (2021) evaluated two BC films as an alternative textile surface for use in the manufacture of clothing prototypes [35]. A combination of experiments was carried out for the production and characterization of biofilms with traditional fabric sewing techniques. The membranes were produced using the bacterium *Gluconacetobacter hansenii* UCP1619 and kombucha. The BC films were then purified, characterized by scanning electron microscopy (SEM), and evaluated for mechanical strength [35].

Two clothing prototypes were developed by combining BC films with a flat fabric composed of 70% linen and 30% polyester to assess the feasibility of the garment for future clothing production using biomaterials. The results showed that the combination of flat fabric with BC-based biomaterials is a viable alternative for manufacturing apparel products. BC application studies in the textile industry are still in their early stages, although they are increasingly attracting the attention of researchers around the world [35].

It is noteworthy that the textile and clothing industry is directly related to complex issues that are contrary to sustainability. The fibers most commonly used in the clothing industry are synthetic and, therefore, non-biodegradable. Thus, BC fibers can be used for textile manufacturing due to their suitable physicochemical and mechanical properties. Thus, efforts have been directed towards improving its characteristics, such as flexibility, especially after dehydration [3].

Kim et al. (2021) carried out an experimental study with the objective of developing bio-leather from BC for sustainability [36]. To increase durability, they used proteins of vegetable origin, such as soy protein isolate (SPI) and mushroom protein (MP), that were physically imprisoned within the BC. As a result, the durability of BC improved after protein trapping. It is noteworthy that the durability of BC trapped with plant-based proteins improved due to the addition of glycerol. Glycerol also contributed to increased water resistance, traction, flexibility, and crease recovery compared to bovine leather. The study confirmed that BC entrapped with proteins and glycerol can be a suitable leather substitute [36].

Innovative studies using BC are also in the space exploration research sectors. According to Kozyrovska et al. (2021), advanced technologies are being developed to support adaptation to extraterrestrial environments and the viability of an independent economy [37]. Microbial communities can be used to support the health of crews in addition to consumable production, waste recycling, and biomining. They can self-renew with few Earth resources, be highly productive by volume, and be highly versatile.

For the authors, an example of this is kombucha, which can be valuable in sustaining the health of crews. BC products offer a wide range of possible functions, from leather-like materials to innovative smart materials during long-term missions and future activities in extraterrestrial settlements. Cellulose production by kombucha is zero waste and may be linked to loops of the bioregenerative life support system (BLSS). Another advantage of kombucha is its ability to mobilize inorganic ions from rocks, which can help feed BLSS from local resources [37].

Further space-related research was carried out. Orlovska et al. (2021) discussed that cellulose may be a macromolecule that is widespread in terrestrial environments, and one of the main architectural components of the microbial biofilm [38]. Cellulose can be considered a biosignature that indicates the presence of microbial life. The researchers presented, for the first time, the characteristics of the BC after long-term spaceflights that were exposed to conditions similar to Mars. After their return to Earth, the samples were reactivated and cultured for 2.5 years to understand if the kombucha cellulosic membrane could be restored [38].

According to the authors, some results could be observed:

(1) The integrity of the cellulose polymer was not significantly altered under conditions similar to those of Mars.
(2) Cellulose production was 1.5 times lower in exposed samples.
(3) The dry cellulose yield of *Komagataeibacter oboediens* reisolated was 1.7 times lower than that of wild type.
(4) There was no significant change in the mechanical properties of the newly synthesized cellulose-based films produced by the BC from the exposed kombucha and *K. oboediens*.
(5) The gene, which encodes the cellulose biosynthesis of *K. oboediens*, was downregulated, and no topological changes were observed in the genes.

Finally, Orlovska et al. (2021) suggested that BC-based film might be a good material to protect microbial communities during space travel [38]. The cellulose produced by kombucha might be suitable for manufacturing consumer goods for extraterrestrial locations.

### 4.3. Production, Sustainability, and Circular Economy: Perspectives, Challenges, and Trends in the Use of Bacterial Cellulose

We live in an era of advanced materials and, currently, the emphasis is on green technologies where the circular economy (CE) is driving innovation and bringing new paradigms [3]. It is known that CE concepts arise from different schools of thought, as can be directly observed by the Ellen MacArthur Foundation [43].

According to Hildebrandt et al. (2021), traditional circular economy concepts focus on waste-to-energy recovery pathways, higher recovery rates in textile recycling, and additional potential for secondary product platforms, among others. Definitions are focusing more from a lifecycle perspective. By incorporating lifecycle management strategies and circular economy design concepts into the lifecycle phases of emerging chains, a more waterfall-oriented end-of-life path is built which promotes the concept of circularity involving more efficient use of agricultural waste [34].

Comino et al. (2021) observed that cereal processing and flour production perform a key role in the Italian agri-food scenario, generating valuable economic income and significantly contributing to the export market. It is known that the European Union has committed itself to the transition to the Circular Economic model. This has led to greater attention to food by-products and waste recovery practices to reduce the amount of food loss, waste, and the disposal costs. One of the solutions found was to carry out research on the opportunity to use wheat powder as a reinforcement for a starch-based biocomposite and as a culture medium for microbial celluloses [40].

Regarding waste management and circular economy, partnerships between sectors can be an excellent solution because through interdisciplinarity, one sector can give a function to the waste generated in another. An example of this is the waste generated during the production of the kombucha probiotic drink, consisting of BC which can be used as a potential biomaterial for textiles, footwear, and other branches of design [5]. The commercialization of the kombucha SCOBY (Symbiotic Consortium of Bacteria and Yeast) by-product can be the sustainable way to transform waste into value-added products [24].

When it comes to environmental concerns, the biopolymer, a renewable and sustainable material, has been studied for several applications. It has been widely used as a raw material for films based on native polymer or in composite formulation, taking advantage of its barrier properties, chemical stability, non-toxicity, biocompatibility, renewability, biodegradability, and mechanical resistance [8].

Other issues related to BC production and its sustainability are also raised, such as purification treatments. The cellulose produced can be purified by different methods based on its large-scale application. The most common is the use of NaOH [38]. This purification is done to remove live cells and compounds from the liquid culture adhering to the film [11]. However, scholars question sustainability considering large-scale manufacturing and the use of large amounts of NaOH. Thus, studies in this direction are also welcome. Cubas et al. (2022) investigated the action of non-thermal plasma technology (NTP) applied in the BC purification process [41]. It should be noted that BC was synthesized using *Gluconacetobacter hansenii*.

The results of the experimental research revealed that after 15 min of NTP treatment, no new membrane appeared when the BC samples were placed back into the culture medium. Thus, the results showed a potential application of NTP in the BCpurification process, which should be researched, as production time has been optimized and can contribute to the mitigation of aggressive chemical residues [41].

Sederavičiūtė et al. (2019) commented that because BCis an ecological, renewable, and organic raw material, it is safe for the human body [2]. It inspired many design projects and caused a lot of attention in the fashion industry. A study carried out by Provin et al. (2021) and collaborators identified that biomaterials, including the use of BC, contribute to Sustainable Development, and to the achievement of the goals of the Sustainable Development Goals (SDGs) such as SDGs 9, 12, and 17 [44]. However, the choice of raw material for bacterial conversion is very important as it affects the environmental footprint of the entire production process and the properties of the final products. Thus, trade-offs between substrate choice and process stability, and product yield, are also important factors that must be evaluated before substrate selection [34].

Gregory et al. (2021) report that the global BCmarket was approximately USD 299 million in 2019, forecasted to reach around USD 777 million in 2027. In the pursuit of sustainable green materials, BC provides an excellent opportunity to address recent trends. As seen earlier, BC is suitable for a wide variety of applications in industrial and biomedical sectors. However, current high production costs and low yields have limited large-scale production of BC and its commercial applications [3].

The BC production process is expensive due to the low productivity of the known strains and the use of extremely expensive fermentation medium, where the defined medium represents about 30% of the total cost. This high expense makes it an obstacle to expansion into large-scale production and other applications. Several studies have recently focused on the use of alternative, natural, and effective nutritional sources, such as agricultural, industrial, and food processing residues as a carbon source, in order to reduce the cost of BC biosynthesis [20,42].

Behera et al. (2022) and Laavanya et al. (2021) comment that the large-scale production of kombucha beverage is seen as a commercially viable and profitable business, while the production of the by-product, that is, the kombucha cellulose biofilm as the main product for use in various applications, still needs to be studied in detail [7,11]. Thus, technical-economic feasibility studies related to the fermentation of kombucha beverage or bacterial nanocellulose arise.

To Sederavičiūtė et al. (2019), although the cultivation methods, reactor designs, growth, and maintenance of kombucha BC are being well studied, large-scale production is often hampered due to the lack of information related to the economic viability of the process. Technical aspects, such as yield, productivity, economic viability, and profitability of the crop must be considered during scale-up and commercialization. The researchers also comment that scientific articles are extremely limited to adapt these materials to the production of large-scale products [2,7].

Laavanya et al. (2021) also noted in their research that there are minimal studies on the large-scale cultivation of kombucha BC. Laavanya et al. (2021) and collaborators experimented with three different ways to increase the yield of the biomaterial [11]:

(1) Static fermentation.
(2) Static and agitated fermentation in two stages.
(3) Fixed-flow bed fermentation.

As per the report, it was observed that the growth of yeast and acetic acid bacteria was maintained throughout the 15 day fermentation period, which resulted in an increased consumption of sugar, acetic acid, and ethanol production. It was then found that with sufficient aeration, the up flow fixed bed fermentation method is comparatively more efficient [11].

**Figure 6:**
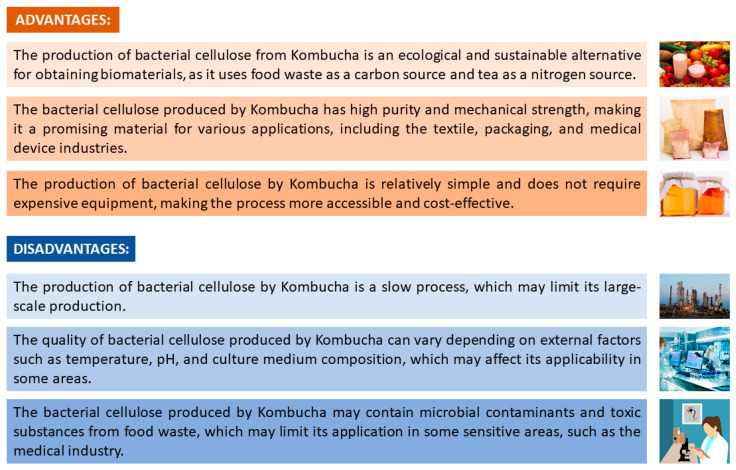
Advantages and disadvantages of bacterial cellulose production through kombucha drink. Source: authors, 2022.

Finally, it should be emphasized that BC has several advantages; however, it also has disadvantages that must be taken into consideration. Figure 6 shows the advantages and disadvantages of BC.

Figure 6 presents three advantages and three disadvantages of producing BC through the kombucha drink. Future research related to BC production is expected to be guided by the improvements and minimization of BC’s disadvantages, aiming for a more sustainable development.

## 5. Final Considerations

This review aimed to organize the existing literature on the advances in biomaterial production using the kombucha beverage and food waste. A total of 42 research and review articles published between 2018 and 2022 were analyzed. Based on the reviewed literature, it was observed that the world is moving towards more sustainable and ecologically friendly materials, such as biomaterials. Important opportunities are emerging in the biotechnology and biomanufacturing sectors.

One of the most identified biomaterials was BC, which can be produced through the probiotic kombucha. Researchers have paid significant attention to BC produced by kombucha due to its excellent mechanical and physicochemical properties, which make it suitable for various applications.

The literature review identified five sectors that have emerged: (a) healthcare, specifically wound healing and burn recovery, although no studies have been found that utilize kombucha in the medical field; (b) packaging, which has become an environmental problem due to population growth and increased consumption; (c) 3D printing, with a focus on waste-free production, biodegradable materials, and the potential to contribute to other sectors such as tissue engineering; (d) textiles as a potential substitute for leather and textiles derived from fossil fuels, such as polyester; and (e) space exploration, as tests of advanced technology with living microorganisms are being developed to support adaptation to extraterrestrial environments and the viability of an independent economy.

The study observed the possibility of reusing food waste to produce BC (using tea from the Camellia sinensis family, rice vinegar, and other sources of nitrogen and carbon, such as fruits, sugarcane, and other foods) to contribute to a Circular Economy. Through a Circular Economy, natural resources can be reused, promoting interdisciplinary dialogue between sectors, and sustainability. This can mitigate negative environmental impacts and help achieve the Sustainable Development Goals (SDGs).

In conclusion, despite the numerous advantages of BC, such as its biodegradability, further studies are needed on large-scale production, economic viability, and its use in certain areas, such as the healthcare sector. Although reusing food waste has been suggested as a way to reduce production costs, further research is necessary. Therefore, this review also suggests advancements in future research to improve BC properties for different sectors and specific treatments for each area. These issues are also interconnected with the process of large-scale commercialization and economic viability.

## Figures and Tables

**Table 1:** Topics covered and the respective references.

Theme	References
Production of bacterial cellulose from food waste	[2,5,7,8,11,14,18,19,20,21,22,23]
Research, sectors, and products: the use of bacterial cellulose and areas of study	[3,6,7,11,12,24,25,26,27,28,29,30,31,32,33,34,35,36,37,38]
Production, sustainability, and circular economy: Perspectives, challenges, and trends in the use of bacterial cellulose	[3,7,11,12,20,24,26,34,39,40,41,42]

Source: authors, 2022.

**Table 2:** Research, objectives, and modes of bacterial cellulose production.

Title	Reference	Objectives and Methods of BC Production
Techno-economic feasibility assessment of bacterial cellulose biofilm production during the Kombucha fermentation process	[7]	Objective: To assess the technical and economic feasibility of a kombucha-based cellulose production facility with an annual capacity of 60 tons using the SuperPro designer. CB production: Carbon source—commercial edible sugar; Nitrogen source—black and green tea.
Recent advances in bacterial cellulose: a low-cost effective production media, optimization strategies, and applications	[20]	Objective: To review the nutritional requirements for maximum CB production, including different optimization strategies for cultivation conditions, and to mention different forms of production, one of which is kombucha. CB production: Carbon source—honey, cornstalk, banana, coconut, among others; Nitrogen source—green, black, and white tea.
Bacterial cellulose: From production optimization to new applications	[21]	Objective: To review the last five years of research on the optimization of BC production and yield, citing kombucha as one of the production methods. CB production: Carbon source—straw, fruit juices, rotten fruit, molasses, wine fermentation broth; Nitrogen source—green tea, kombucha tea, black tea, rooibos tea, and corn silk tea.
Optimization and physicochemical characterization of bacterial cellulose by Komagataeibacter nataicola and Komagataeibacter maltaceti strains isolated from grape, thorn apple, and apple vinegars	[23]	Objective: To optimize and characterize the physicochemical properties of bacterial cellulose by the strains *Komagataeibacter nataicola* and *Komagataeibacter maltaceti* isolated from grape, hawthorn, and apple cider vinegar, citing kombucha as one of the production methods. CB production: The bacteria were isolated from grape, hawthorn, and apple cider vinegar and cultured until they produced pure CB. However, kombucha is cited as a method of CB production.
Current challenges, applications and future perspectives of SCOBY cellulose of Kombucha fermentation	[11]	Objective: To review the microbial ecology present in kombucha tea fermentation, the production of extracellular polysaccharides (cellulose) by the bacteria, methods of SCOBY cultivation, composition, structure, and characteristics of obtained cellulose biofilms. CB production: Carbon source—sucrose; Nitrogen source—green tea, black tea, and oolong tea.
Production of kombucha-like beverage and bacterial cellulose by acerola byproduct as raw material	[18]	Objective: To use acerola by-product as a new raw material for the production of kombucha-type beverage and bacterial cellulose. CB production: Carbon source—acerola, fructose, and glucose; Nitrogen source—green tea.
Characterization of bacterial cellulose produced by *Acetobacter pasteurianus* MGC-N8819 utilizing lotus rhizome	[19]	Objective: To isolate three new strains from traditional rice vinegar confirmed to be capable of producing BC, and one of the production methods is kombucha. CB production: Carbon source—lotus rhizome; Nitrogen source—rice vinegar and kombucha tea.
Circular economy for fashion industry: Use of waste from the food industry for the production of biotextiles	[39]	Objective: To highlight the reuse of food industry waste for the manufacture of a new value-added textile product known as CB, with one of the production methods being kombucha. Production of CB: Carbon source—food residues, including saccharified food residues, grape medium, pineapple juice, grape marc; Nitrogen source—green tea, black tea.
Cost-effective production of bacterial cellulose using acidic food industry by-products	[22]	Objective: To reduce the cost of obtaining bacterial cellulose by using acidic byproducts from alcohol and dairy industries without any pre-treatment or addition of other nitrogen sources. The bacterial culture of *G. sucrofermentans* B-11267 used in this study was isolated from kombucha tea. Production of CB: Carbon source—wheat straw, spruce hydrolysate, wood hot water extracts, pineapple agroindustrial residues, fruit juices, rotten fruit, cotton-based waste textiles, molasses, waste from the dairy industry, wine fermentation waste broth, wastewater of candied jujube processing industry, and waste and by-product streams from biodiesel and confectionery industries and acetone-butanol-ethanol fermentation.
Effect of pretreatment procedure on properties of Kombucha fermented bacterial cellulose membrane	[2]	Objective: To analyze the structure and properties of bacterial cellulose membrane (BCM). Production of CB: Carbon source—sucrose; Nitrogen source—green tea.
Kombucha Tea By-Product as Source of Novel Materials: Formulation and Characterization of Films	[12]	Objective: To develop new materials based on the integral byproduct of kombucha tea using floating and intentionally submerged biomass discs. Production of CB: Carbon source—sucrose; Nitrogen source—black tea.
*Komagataeibacter rhaeticus* grown in sugarcane molasses-supplemented culture medium as a strategy for enhancing bacterial cellulose production	[14]	Objective: To use *Komagataeibacter rhaeticus*, a bacteria isolated from kombucha tea, to produce bacterial cellulose (BC) using sugar cane molasses. Production of CB: Carbon source—sugar cane molasses; Nitrogen source—green tea.

Source: author, 2022.

**Table 3:** Patents for bacterial cellulose production using kombucha.

Title	Publication Year/Status	Current Assignee	Patent Registration/Country
Method for preparing bacterial cellulose by using wheat straw	2012/Active	Donghua University	CN101985641B/China
Method and device for preparing bacterial cellulose composite material quickly on large scale	2014/Active	Donghua University	CN102533904B/China
Preparation method of Bacterial Cellulose (BC) with carbon source of inulin	2014/Active	Donghua University	CN102242166B/China
Method for preparing bacterial cellulose by tuberous raw materials	2014/Active	Donghua University	CN102703543B/China
*Komagataeibacter rhaeticus p 1463* produtor de celulose bacteriana	2016/Active	Latvijas Universitate	EP3121265B1/ Latvia
A kind of method that Bacterial cellulose is prepared as carbon source with Jerusalem artichoke	2017/Active	Donghua University	CN102250983B/China

Source Authors, 2022.

## Data Availability

The data are fully included in the article.

## Appendix A

**Table A1:** Specific details of the research and review articles used to write this review.

**Title**	**Reference**	**Country**	**Research Area**	**Citation**
Optimization and physicochemical characterization of enhanced microbial cellulose production with a new Kombucha consortium	[27]	Turkey	Biology	8
Statistical optimization and characterization of bacterial cellulose produced by isolated thermophilic *Bacillus licheniformis* strain ZBT2	[30]	India	Biotechnology; Biochemistry	17
Techno-economic feasibility assessment of bacterial cellulose biofilm production during the Kombucha fermentation process	[7]	India	Biotechnology; Medical Engineering	6
Aflatoxin B1 degradation by microorganisms isolated from Kombucha culture	[10]	Tunisia and Saudi Arabia	Pharmacy; Engineering	28
Do-it-yourself approach applied to the valorisation of a wheat milling industry’s by-product for producing bio-based material	[40]	Italy	Environment, Land, and Infrastructure Engineering; Architecture and Design	4
Bacterial cellulose: characterization of a biomaterial for apparel products application	[35]	Brazil	Biotechnology; Fashion Design; Design; Technology and innovation	0
Application of non-thermal plasma as an alternative for purification of bacterial cellulose membranes	[41]	Brazil	Environmental Science; Chemical Engineering	0
Recent advances in bacterial cellulose: a low-cost effective production media, optimization strategies and applications	[20]	Egypt and USA	Genetic Engineering and Biotechnology; Biology and Health Sciences	0
Bacterial cellulose: From production optimization to new applications	[21]	Brazil	Food Engineering; Chemistry and Biology	50
Bacterial cellulose: A smart biomaterial with diverse applications	[3]	UK and France	Materials Science and Engineering	35
Optimization and physicochemical characterization of bacterial cellulose by *Komagataeibacter nataicola* and *Komagataeibacter maltaceti* strains isolated from grape, thorn apple and apple vinegars	[23]	Poland and Turkey	Pharmacy; Biotechnology	0
Biotechnological production of cellulose by acetic acid bacteria: current state and perspectives	[31]	Italy	Life Sciences; Agricultural, Food and Environmental Sciences	75
Characterization of nanocellulose production by strains of *Komagataeibacter* sp. isolated from organic waste and Kombucha	[4]	India	Biology; Advanced Analytical Sciences	9
The remarkable three-dimensional network structure of bacterial cellulose for tissue engineering applications	[6]	Malaysia and Italy	Dentistry; Engineering; and Architecture	50
The circularity of potential bio-textile production routes: Comparing life cycle impacts of bio-based materials used within the manufacturing of selected leather substitutes	[34]	Germany	Environmental Research; Process Development, Peat and Natural Materials	16
Development of plant extract impregnated bacterial cellulose as a green antimicrobial composite for potential biomedical applications	[16]	Saudi Arabia	Chemistry; Engineering; Advanced Materials	1
Comparative study on the physical entrapment of soy and mushroom proteins on the durability of bacterial cellulose bio-leather	[36]	South Korea	Clothing and Textiles; Fashion and Clothing	9
Emerging challenges for the agro-industrial food waste utilization: A review on food waste biorefinery	[15]	India, Saudi Arabia and Finland	Medical and Technical Sciences; Chemistry; Advanced Materials Science; Biotechnology and Engineering	18
To Other Planets With Upgraded Millennial Kombucha in Rhythms of Sustainability and Health Support	[37]	Ukraine South Africa, Brazil, India, Germany, Italy, Poland and Netherlands	Molecular Biology and Genetics; Bioinformatics and Computational Biology, Genetics and Microbiology; Astrobiology	5
Current challenges, applications and future perspectives of SCOBY cellulose of Kombucha fermentation	[11]	India	Biotechnology and Medical Engineering	51
Production of kombucha-like beverage and bacterial cellulose by acerola byproduct as raw material	[18]	Brazil	Chemical and Food Engineering Engineering; Molecular Biology and Biotechnology	26
*Komagataeibacter rhaeticus* grown in sugarcane molasses-supplemented culture medium as a strategy for enhancing bacterial cellulose production	[14]	Brazil e Spain	Advanced Materials; Chemical and Environmental Engineering	56
Wound healing and anti-inflammatory effects of bacterial cellulose coated with *Pistacia atlantica* fruit oil	[28]	Iran	Pharmacy; Medical Sciences	2
Bacterial cellulose: A promising biopolymer with interesting properties and applications	[29]	India	Materials Science; Polymer Science and Technology	0
Characterization of bacterial cellulose produced by *Acetobacter pasteurianus* MGC-N8819 utilizing lotus rhizome	[19]	China	Food Science and Biotechnology	0
Bacterial Cellulose Retains Robustness but Its Synthesis Declines after Exposure to a Mars-like Environment Simulated Outside the International Space Station	[38]	Ukraine, Serbia, Brazil, India, Germany and USA	Molecular Biology and Genetics; Physics; Nuclear Sciences; Bioorganic Chemistry and Petrochemistry; Veterinary Medicine	14
Symbiotic culture of nanocellulose pellicle: A potential matrix for 3D bioprinting	[33]	India and South Korea	Functional; Innovative and Smart Textiles; Chemical and Biomolecular Engineering; Biomedical Engineering and Biomaterials	14
Machine learning prediction of SCOBY cellulose yield from Kombucha tea fermentation	[24]	India	Biotechnology and Medical Engineering; Food Process Engineering	2
Textile industry and environment: can the use of bacterial cellulose in the manufacture of biotextiles contribute to the sector?	[5]	Brazil	Environmental Science; Clothing and Fashion Design	2
Circular economy for fashion industry: Use of waste from the food industry for the production of biotextiles	[39]	Brazil and Portugal	Environmental Science; Fiber Materials and Environmental Technologies	32
New materials for clothing: Rethinking possibilities through a sustainability approach—A review	[44]	Brazil	Environmental Science	10
Cost-effective production of bacterial cellulose using acidic food industry by-products	[22]	Russian Federation	Biotechnology and Biology	153
Effect of pretreatment procedure on properties of Kombucha fermented bacterial cellulose membrane	[2]	Lithuania	Mechanical Engineering and Design	24
Influence of drying temperature on tensile and bursting strength of bacterial cellulose biofilm	[26]	Lithuania e Latvia	Mechanical Engineering and Design; Material Sciences and Applied Chemistry	5
Static intermittent fed-batch production of bacterial nanocellulose from black tea and its modification using chitosan to develop antibacterial green packaging material	[32]	India	Industrial Research and Development	20
Developments in bioprocess for bacterial cellulose production	[1]	Taiwan and India	Marine Environmental Engineering; Integrative Medicine; Biotechnology; Microbiology	7
Cellulosic biofilm formation of *Komagataeibacter* in kombucha at oil-water interfaces	[25]	Austria	Nanobiotechnology	2
Bacterial cellulose films production by Kombucha symbiotic community cultured on different herbal infusions	[8]	Argentina	Science and Technology	10
Kombucha Tea By-product as Source of Novel Materials: Formulation and Characterization of Films	[12]	Argentina	Science and Technology	19
Microbiological and sensory characterization of kombucha SCOBY for culinary applications	[13]	Spain	Gastronomy; Biology; Nanoscience and Nanotechnology	7
Shedding Light on the Formation and Structure of Kombucha Biofilm Using Two-Photon Fluorescence Microscopy	[9]	France	Microbiology	5
Physicochemical properties of bacterial cellulose obtained from different Kombucha fermentation conditions	[42]	France	Chemical Engineering	4

## References

[1] Singhania R.R., Patel A.K., Tseng Y.S., Kumar V., Chen C.W., Haldar D., Saini J.K., Dong C.-D. (2022). Developments in Bioprocess for Bacterial Cellulose Production. Bioresour. Technol..

[2] Sederavičiūtė F., Bekampienė P., Domskienė J. (2019). Effect of Pretreatment Procedure on Properties of Kombucha Fermented Bacterial Cellulose Membrane. Polym. Test..

[3] Gregory D.A., Tripathi L., Fricker A.T.R., Asare E., Orlando I., Raghavendran V., Roy I. (2021). Bacterial Cellulose: A Smart Biomaterial with Diverse Applications. Mater. Sci. Eng. R Rep..

[4] Gupte Y., Kulkarni A., Raut B., Sarkar P., Choudhury R., Chawande A., Kumar G.R.K., Bhadra B., Satapathy A., Das G. (2021). Characterization of Nanocellulose Production by Strains of *Komagataeibacter* sp. Isolated from Organic Waste and Kombucha. Carbohydr. Polym..

[5] Provin A.P., Cubas A.L.V., Dutra A.R.d.A., Schulte N.K. (2021). Textile Industry and Environment: Can the Use of Bacterial Cellulose in the Manufacture of Biotextiles Contribute to the Sector?. Clean Technol. Environ. Policy.

[6] Halib N., Ahmad I., Grassi M., Grassi G. (2019). The Remarkable Three-Dimensional Network Structure of Bacterial Cellulose for Tissue Engineering Applications. Int. J. Pharm..

[7] Behera B., Laavanya D., Balasubramanian P. (2022). Techno-Economic Feasibility Assessment of Bacterial Cellulose Biofilm Production during the Kombucha Fermentation Process. Bioresour. Technol..

[8] Tapias Y.A.R., Di Monte M.V., Peltzer M.A., Salvay A.G. (2022). Bacterial Cellulose Films Production by Kombucha Symbiotic Community Cultured on Different Herbal Infusions. Food Chem..

[9] Tran T., Grandvalet C., Winckler P., Verdier F., Martin A., Alexandre H., Tourdot-Maréchal R. (2021). Shedding Light on the Formation and Structure of Kombucha Biofilm Using Two-Photon Fluorescence Microscopy. Front. Microbiol..

[10] Ben Taheur F., Mansour C., Ben Jeddou K., Machreki Y., Kouidhi B., Abdulhakim J.A., Chaieb K. (2020). Aflatoxin B1 Degradation by Microorganisms Isolated from Kombucha Culture. Toxicon.

[11] Laavanya D., Shirkole S., Balasubramanian P. (2021). Current Challenges, Applications and Future Perspectives of SCOBY Cellulose of Kombucha Fermentation. J. Clean. Prod..

[12] Tapias Y.A.R., Peltzer M.A., Delgado J.F., Salvay A.G. (2020). Kombucha Tea By-Product as Source of Novel Materials: Formulation and Characterization of Films. Food Bioprocess Technol..

[13] Torán-Pereg P., del Noval B., Valenzuela S., Martinez J., Prado D., Perisé R., Arboleya J.C. (2021). Microbiological and Sensory Characterization of Kombucha SCOBY for Culinary Applications. Int. J. Gastron. Food Sci..

[14] Machado R.T.A., Meneguin A.B., Sábio R.M., Franco D.F., Antonio S.G., Gutierrez J., Tercjak A., Berretta A.A., Ribeiro S.J.L., Lazarini S.C. (2018). *Komagataeibacter rhaeticus* Grown in Sugarcane Molasses-Supplemented Culture Medium as a Strategy for Enhancing Bacterial Cellulose Production. Ind. Crops Prod..

[15] Kumar V., Sharma N., Umesh M., Selvaraj M., Al-Shehri B.M., Chakraborty P., Duhan L., Sharma S., Pasrija R., Awasthi M.K. (2022). Emerging Challenges for the Agro-Industrial Food Waste Utilization: A Review on Food Waste Biorefinery. Bioresour. Technol..

[16] Kamal T., Ul-Islam M., Khan S.B., Bakhsh E.M., Chani M.T.S. (2022). Development of Plant Extract Impregnated Bacterial Cellulose as a Green Antimicrobial Composite for Potential Biomedical Applications. Ind. Crops Prod..

[17] (2015). United Nation Development Programme (UNDP) Sustainable Development Goals. https://www.undp.org/.

[18] Leonarski E., Cesca K., Zanella E., Stambuk B.U., de Oliveira D., Poletto P. (2021). Production of Kombucha-like Beverage and Bacterial Cellulose by Acerola Byproduct as Raw Material. LWT.

[19] Nie W., Zheng X., Feng W., Liu Y., Li Y., Liang X. (2022). Characterization of Bacterial Cellulose Produced by Acetobacter Pasteurianus MGC-N8819 Utilizing Lotus Rhizome. LWT.

[20] El-Gendi H., Tarek T.H., Ray J.B., Saleh A.K. (2022). Recent Advances in Bacterial Cellulose: A Low-Cost Effective Production Media, Optimization Strategies and Applications. Cellulose.

[21] Fernandes I.d.A.A., Pedro A.C., Ribeiro V.R., Bortolini D.G., Ozaki M.S.C., Maciel G.M., Haminiuk C.W.I. (2020). Bacterial Cellulose: From Production Optimization to New Applications. Int. J. Biol. Macromol..

[22] Revin V., Liyaskina E., Nazarkina M., Bogatyreva A., Shchankin M. (2018). Cost-Effective Production of Bacterial Cellulose Using Acidic Food Industry by-Products. Braz. J. Microbiol..

[23] Greser A.B., Avcioglu N.H. (2022). Optimization and Physicochemical Characterization of Bacterial Cellulose by *Komagataeibacter nataicola* and *Komagataeibacter maltaceti* Strains Isolated from Grape, Thorn Apple and Apple Vinegars. Arch. Microbiol..

[24] Priyadharshini T., Nageshwari K., Vimaladhasan S., Parag Prakash S., Balasubramanian P. (2022). Machine Learning Prediction of SCOBY Cellulose Yield from Kombucha Tea Fermentation. Bioresour. Technol. Rep..

[25] Subbiahdoss G., Osmen S., Reimhult E. (2022). Cellulosic Biofilm Formation of *Komagataeibacter* in Kombucha at Oil-Water Interfaces. Biofilm.

[26] Sederavičiūtė F., Domskienė J., Baltina I. (2019). Influence of Drying Temperature on Tensile and Bursting Strength of Bacterial Cellulose Biofilm. Medziagotyra.

[27] Avcioglu N.H., Birben M., Bilkay I.S. (2021). Optimization and physicochemical characterization of enhanced microbial cellulose production with a new Kombucha consortium. Process Biochem..

[28] Mirmohammadsadegh N., Shakoori M., Moghaddam H.N., Farhadi R., Shahverdi A.R., Amin M. (2022). Wound Healing and Anti-Inflammatory Effects of Bacterial Cellulose Coated with *Pistacia atlantica* Fruit Oil. DARU J. Pharm. Sci..

[29] Navya P.V., Gayathri V., Samanta D., Sampath S. (2022). Macromolecules Bacterial Cellulose: A Promising Biopolymer with Interesting Properties and Applications. Int. J. Biol. Macromol..

[30] Bagewadi Z.K., Bhavikatti J.S., Muddapur U.M., Yaraguppi D.A., Mulla S.I. (2020). Statistical Optimization and Characterization of Bacterial Cellulose Produced by Isolated Thermophilic *Bacillus licheniformis* Strain ZBT2. Carbohydr. Res..

[31] Gullo M., China S.L., Falcone P., Giudici P. (2018). Biotechnological Production of Cellulose by Acetic Acid Bacteria: Current State and Perspectives. Appl. Microbiol. Biotechnol..

[32] Sharma C., Bhardwaj N.K., Pathak P. (2021). Static Intermittent Fed-Batch Production of Bacterial Nanocellulose from Black Tea and Its Modification Using Chitosan to Develop Antibacterial Green Packaging Material. J. Clean. Prod..

[33] Pillai M.M., Tran H.N., Sathishkumar G., Manimekalai K., Yoon J.H., Lim D.Y., Noh I., Bhattacharyya A. (2021). Symbiotic Culture of Nanocellulose Pellicle: A Potential Matrix for 3D Bioprinting. Mater. Sci. Eng. C.

[34] Hildebrandt J., Thrän D., Bezama A. (2021). The Circularity of Potential Bio-Textile Production Routes: Comparing Life Cycle Impacts of Bio-Based Materials Used within the Manufacturing of Selected Leather Substitutes. J. Clean. Prod..

[35] Costa A.F.D.S., Rocha M.A.V., Fenrnandes L.M.A., Queiroz J.A., Agra A.C.M.G., Amorim J.D.P., Sarubbo L.A., Sarubbo L.A. (2021). Bacterial Cellulose: Characterization of a Biomaterial for Apparel Products Application. Res. J. Text. Appar..

[36] Kim H.H.R., Song J.E., Kim H.H.R. (2021). Comparative Study on the Physical Entrapment of Soy and Mushroom Proteins on the Durability of Bacterial Cellulose Bio-leather. Cellulose.

[37] Kozyrovska N., Reva O., Podolich O., Kukharenko O., Orlovska I., Terzova V., Zubova G., Trovatti Uetanabaro A.P., Góes-Neto A., Azevedo V. (2021). To Other Planets with Upgraded Millennial Kombucha in Rhythms of Sustainability and Health Support. Front. Astron. Space Sci..

[38] Orlovska I., Podolich O., Kukharenko O., Zaets I., Reva O., Khirunenko L., Zmejkoski D., Rogalsky S., Barh D., Tiwari S. (2021). Bacterial Cellulose Retains Robustness but Its Synthesis Declines after Exposure to a Mars-like Environment Simulated Outside the International Space Station. Astrobiology.

[39] Provin A.P., Dutra A.R.d.A., Gouveia I.C., Cubas A.L.V. (2021). Circular Economy for Fashion Industry: Use of Waste from the Food Industry for the Production of Biotextiles. Technol. Forecast. Soc. Chang..

[40] Comino E., Dominici L., Perozzi D. (2021). Do-It-Yourself Approach Applied to the Valorisation of a Wheat Milling Industry’s by-Product for Producing Bio-Based Material. J. Clean. Prod..

[41] Cubas A.L.V., Tayane R., Oliveira D.D., Cesca K. (2022). Application of Non-Thermal Plasma as an Alternative for Purification of Bacterial Cellulose Membranes. Sustain. Chem. Pharm..

[42] Villarreal-Soto S.A., Bouajila J., Beaufort S., Bonneaud D., Souchard J.P., Taillandier P. (2021). Physicochemical Properties of Bacterial Cellulose Obtained from Different Kombucha Fermentation Conditions. J. Vinyl Addit. Technol..

[43] EMF Ellen Macarthur Foundation How to Build a Circular Economy|Ellen MacArthur Foundation. https://ellenmacarthurfoundation.org/.

[44] Provin A.P., Regina de Aguiar Dutra A., Machado M.M., Vieira Cubas A.L. (2021). New Materials for Clothing: Rethinking Possibilities through a Sustainability Approach—A Review. J. Clean. Prod..

